# Recurrent Takotsubo syndrome complicated with ischemic enteritis successfully treated by hydration: a case report

**DOI:** 10.1186/s13256-021-03194-6

**Published:** 2021-12-19

**Authors:** Shunsuke Todani, Mao Takahashi

**Affiliations:** Cardiovascular Center, Toho University, Sakura Hospital, 564-1 Shimoshizu, Sakura, Chiba 285-8741 Japan

**Keywords:** Takotsubo syndrome, Vasospastic angina, Hydration, Early recovery, Case report

## Abstract

**Background:**

Takotsubo syndrome is a sudden and an acute form of transient cardiac dysfunction, triggered by mental and physical stress. The treatment for Takotsubo syndrome is not well understood and is incompletely established. Takotsubo syndrome is partly thought to be caused by coronary ischemia under sympathetic nerve activation.

**Case presentation:**

We report the case of an 80-year-old Japanese woman with recurrent Takotsubo syndrome complicated with ischemic enteritis. In this case, abdominal pain and dehydration due to ischemic enteritis is thought to have triggered Takotsubo syndrome. Her life was saved with rapid, adequate intravenous hydration. She was diagnosed with coronary vasospastic angina using coronary angiography on her second admission. This case highlights the potential of adequate intravenous hydration in increasing coronary blood flow. In our case, it should be noted that pulmonary congestion was mild and may have improved Takotsubo syndrome without the use of diuretics.

**Conclusion:**

Adequate hydration must be considered for prompt improvement of cardiac function in Takotsubo syndrome. Replenishment of fluid to increase coronary blood flow, improvement of heart load without exacerbating heart failure, and stabilization of circulation dynamics can help treat patients with Takotsubo syndrome without using diuretics.

## Background

Takotsubo syndrome (TTS) is a recently recognized condition characterized by transient cardiac dysfunction and ventricular ballooning. Many cases of TTS are known to recover from acute heart failure, but often recur [1]. The precise pathophysiological mechanisms behind TTS are incompletely understood. However, considerable evidence suggests that sympathetic stimulation is central to its pathogenesis [1]. Patients with TTS exhibit characteristic temporary cardiac dysfunction triggered by mental and physical stress [2–4]. Coronary ischemia is believed to be among the factors involved in the pathogenesis of TTS [2]. Adequate hydration may be effective for the acute treatment of TTS by increasing the coronary flow. However, this hypothesis has not yet been clarified. Here, we report a case where TTS was effectively managed using hydration therapy.

## Case presentation

**Fig. 1:**
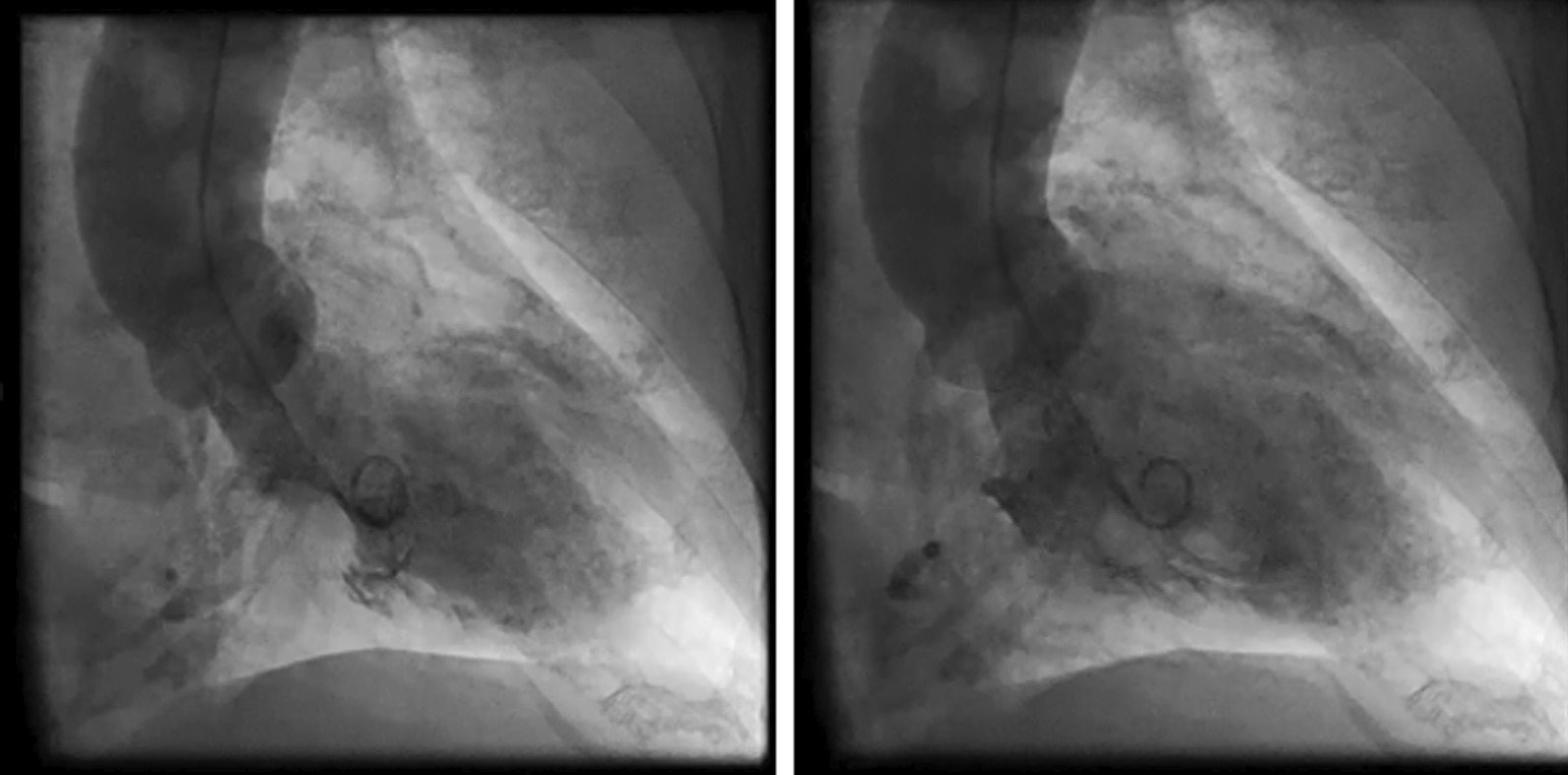
“Four months before hospitalization, the patient underwent left ventriculography and was diagnosed with Takotsubo syndrome.” Coronary angiography 4 months previously showed no significant stenosis. Left ventriculography showed wall motion abnormality centered on the left central ventricle, with ballooning of the apical portion.

An 80-year-old Japanese woman presented with a history of TTS complicated by ischemic enteritis (Fig. 1). She was previously admitted to our hospital, where she presented with bloody stools due to ischemic enteritis, and was treated with hydration of 1500–2500 mL/day and dobutamine. The patient was subsequently discharged without cardioprotective drugs. She was married and had one daughter. She had no family history of cardiovascular disease. She used to cook at a nursing home but she retired. She had smoking history and a drinking habit. She presented to our hospital with upper abdominal pain and bloody stools, 4 months after her first hospital admission for TTS.

At the examination, her general condition was good, she was conscious alert, with a temperature of 36.6 °C. Her height and weight were 148 cm and 42 kg (body mass index 19), respectively. Her blood pressure was 114/80 mmHg, heart rate was 90 bpm, and arterial oxygen saturation on room air was 97%. Her abdominal pain improved upon admission. Her abdomen was flat, soft, tender, and had good gurgling,however, digital rectal examination showed blood on the examining finger, indicating a possible relapse of ischemic enteritis. Physical examination showed a regular cardiac rhythm with normal S1 and S2, no detectable murmurs, and clear lungs. Although the patient did not experience chest pain, her electrocardiogram revealed negative T waves in many leads (I, II, III, aVL, aVF, V2, V3, V4, V5, V6). Blood examination showed that her brain natriuretic peptide (BNP) and troponin I levels had risen to 1578 pg/mL (healthy upper limit 18.4 pg/mL) and 357.2 pg/mL (healthy upper limit 15.6 pg/mL), respectively. Her blood urea nitrogen (BUN) and creatinine levels had risen to 26.1 mg/dL (healthy upper limit 20.0 mg/dL) and 0.87 mg/dL (healthy upper limit 0.79 mg/dL), respectively. There were no findings of liver dysfunction. Echocardiography showed wall motion abnormality centered on the left central ventricle, with ballooning of the apical portion. We suspected the recurrence of TTS. We believe that the abdominal pain and dehydration due to ischemic enteritis may have contributed to the development of TTS.

**Fig. 2:**
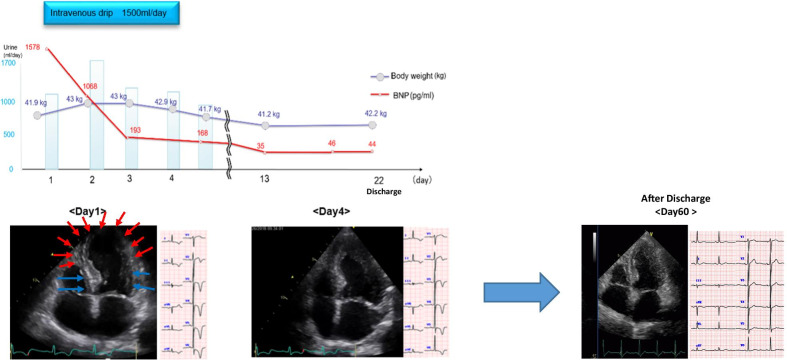
*“Clinical course”.* The electrocardiogram revealed negative T waves in many leads (I, II, III, aVL, aVF, V2, V3, V4, V5, V6). Blood examination showed that her brain natriuretic peptide (BNP) levels had risen to 1578 pg/mL (healthy upper limit 18.4 pg/mL). Her blood urea nitrogen (BUN) and creatinine levels had risen to 26.1 mg/dL (healthy upper limit 20.0 mg/dL) and 0.87 mg/dL (healthy upper limit 0.79 mg/dL), respectively. Echocardiography showed wall motion abnormality centered on the left central ventricle, with ballooning of the apical portion. We suspected the recurrence of TTS. As a treatment, we gave the patient a small amount of oxygen (2 L/min). She also received 10,000 units/day of continuous intravenous heparin for 2 days to prevent left ventricular thrombosis and fluid replacement of 1500 mL/day to treat TTS. Although her body weight increased temporarily, the urine volume was normal, oxygenation was stable, and exacerbation of heart failure was not observed. The BNP level also showed a downward trend; hence, diuretics were not administered and hydration was continued. The BNP level and myocardial wall motion were normalized on the fourth day after admission. She was discharged on the 22nd day. The negative T-wave on the electrocardiogram normalized on the 60th day after discharge

As a treatment, we gave the patient a small amount of oxygen (2 L/min). She also received 10,000 units/day of continuous intravenous heparin for 2 days to prevent left ventricular thrombosis, and fluid replacement of 1500 mL/day to treat TTS (Fig. 2). Although her body weight increased temporarily, the urine volume was normal, oxygenation was stable, and exacerbation of heart failure was not observed. The BNP level also showed a downward trend; hence, diuretics were not administered and hydration was continued. The BNP level and myocardial wall motion were normalized on the fourth day after admission (Fig. 2).

For differentiation of ischemic heart disease, a coronary angiography and an acetylcholine provocation test were conducted on the 22nd day after admission. Although no significant stenosis was found in the coronary artery, the acetylcholine provocation test revealed a considerable multivessel coronary spasm in the left coronary artery, suggesting coronary vasospastic angina pectoris associated with TTS, which was treated using β-blockers, calcium channel blockers, and nicorandil. She was discharged on the 22nd day. The negative T-wave on the electrocardiogram normalized on the 60th day after discharge. (Fig. 2). She was also treated in gastroenterology and psychiatry, and was prescribed probiotics and anxiolytics. Her ischemic enteritis remained stable without abdominal pain. Subsequent follow-up showed no recurrence of TTS over 3 years.

## Discussion and conclusions

**Fig. 3:**
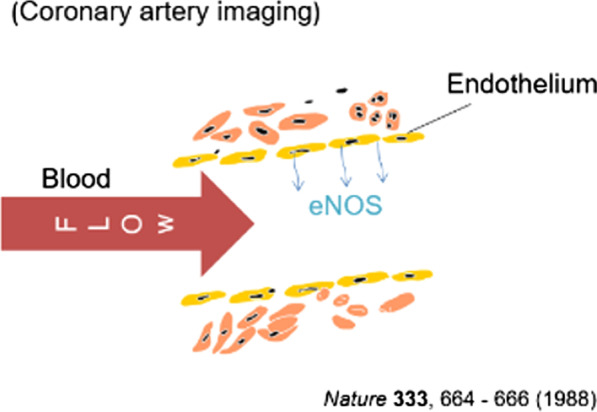
“Hypothesis mechanism of Takotsubo syndrome.” Hydration is known to increase the rate of coronary blood flow, and coronary shear stress induces coronary vasodilation via endothelial type nitrous oxide synthase (eNOS). Proper hydration was performed to increase coronary blood flow as an acute treatment for Takotsubo syndrome

Herein, we described a case of TTS in an 80-year-old woman. Acute treatment involved administering oxygen and appropriate replacement fluids instead of diuretics. TTS with a poor prognosis have also been reported in the acute phase [1]. Oxygen administration reportedly causes vasodilation, which was found to be effective in this case [5]. In addition, hydration is known to increase the rate of coronary blood flow, and coronary shear stress induces coronary vasodilation via endothelial type nitrous oxide synthase (eNOS) [6] (Fig. 3). The production of eNOS by vascular endothelial cells increases due to the administration of nitrate drugs or calcium antagonists; however, these drugs cause outflow tract strictures and low blood pressure, increasing the risk of worsening TTS. Instead of these agents, the coronary artery can be expanded by hydration, leading to early improvement in cardiac function [6]. Careful observation of the patient’s general condition is necessary while adjusting the volume of the replacement fluid, with particular attention to the deterioration of cardiac function. It is essential to know that therapies for specific disease conditions, including catecholamines, intraaortic balloon pumping, antiarrhythmic drugs, and anticoagulants, may adversely affect other disease states.

We did not administer calcium antagonists to avoid worsening the outflow tract stricture during the acute phase of TTS, which is when cardiac hypercontractility is observed. However, following improvement of the excessive contraction of the base in the chronic phase, calcium antagonists were administered in conjunction with nicorandil to treat the coronary vasospastic angina [7]. The use of β-blockers alone did not lead to an acute phase as it may have worsened the coronary spasm-associated angina. β-blockers were introduced in the chronic phase with coronary dilatory drug. Adequate hydration and coronary vasodilator improved TTS immediately and it did not recur. Therefore, our treatment appears to be effective in both the acute and chronic phases.

Herein, we report a case in which TTS occurred repeatedly, following abdominal pain and dehydration due to ischemic enteritis. Our treatment of TTS in the acute phase centered on replenishment of fluid to increase coronary blood flow, improve heart load without exacerbating heart failure, and stabilize circulation dynamics. We recommend that clinicians consider treating TTS using hydration rather than diuretics in patients with dehydration.

## Abbreviations

APSC: Asian Pacific Society of Cardiology
BNP: Brain natriuretic peptide
TTS: Takotsubo syndrome
